# Sandstrahlverletzung der Hornhaut? Ein unerwarteter Hornhautbefund

**DOI:** 10.1007/s00347-021-01338-z

**Published:** 2021-02-19

**Authors:** Christiane Kesper, Anja Viestenz, Arne Viestenz

**Affiliations:** Klinik und Poliklinik für Augenheilkunde, Universitätsklinikum Halle, Martin-Luther-Universität Halle-Wittenberg, Ernst-Grube-Str. 40, 06120 Halle (Saale), Deutschland

## Anamnese

Ein 45-jähriger Patient wurde durch Sandpartikel bei Arbeiten in der Häuslichkeit mit einem Sandstrahler zum Säubern einer Oberfläche im Gesicht und an beiden Augen getroffen. Zu dem verwendeten Gerät konnten seitens des Patienten keine genaueren Angaben gemacht werden. Eine Schutzbrille sei nicht getragen worden. Er klagte über ein Fremdkörpergefühl sowie Verschwommensehen. Anamnestisch bestünde eine Amblyopie des rechten Auges, ansonsten seien keine ophthalmologischen Besonderheiten bekannt. Ein anderes Trauma sei nicht erinnerlich und die Familienanamnese unauffällig. Allgemeine Erkrankungen sowie Allergien werden ebenfalls verneint.

## Klinischer Befund und Diagnostik

**Figure 1:**
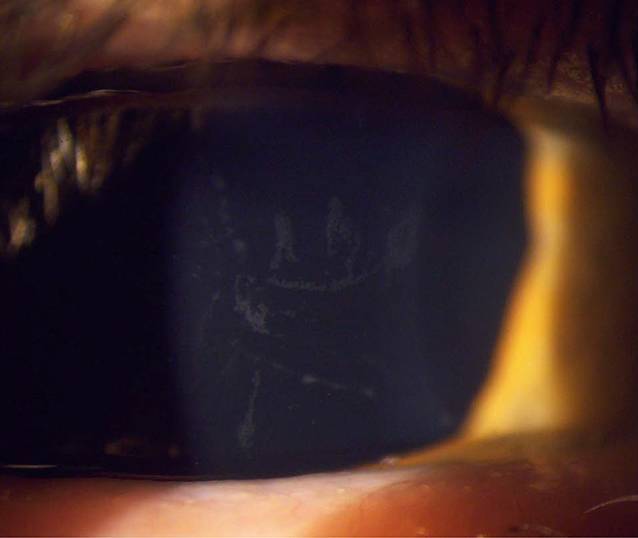

**Figure 2:**
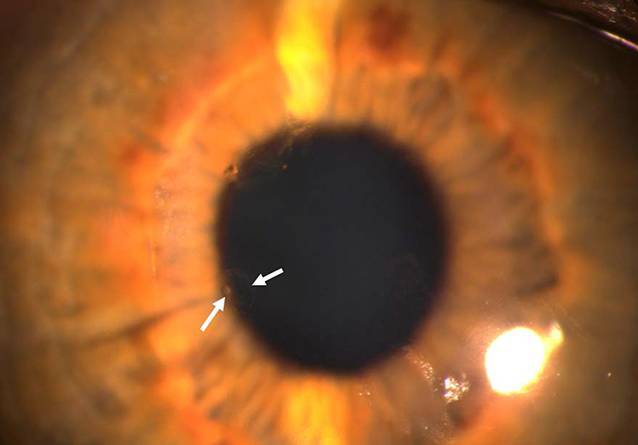

**Figure 3:**
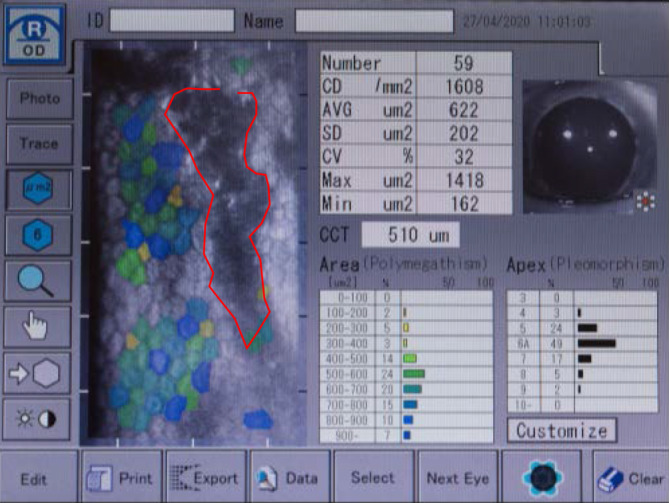

**Figure 4:**
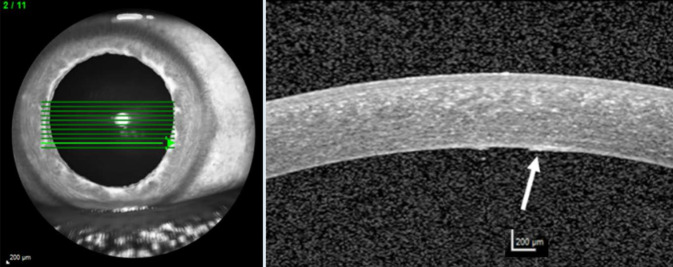

Der bestkorrigiere Visus betrug rechts 0,6 und links 1,0. Spaltlampenmikroskopisch zeigte sich noch wenig Sandmaterial an den Zilien. Die Hornhautoberfläche war aufgeraut und Fluoreszein-positiv gestippt. Es zeigten sich auch beim Ektropionieren keine weiteren Fremdkörper. Am rechten Auge fiel zentral ein endotheliales Ödem auf, welches in etwa 2 mm breit und 7 mm hoch war und nasal und temporal von einem Descemet-Riss begrenzt wurde (Abb. 1). Am linken Auge zeigten sich 3 parazentral gelegene hufeisenförmige Endotheldefekte von einer Größe von je etwa 0,5 mm (Abb. 2). Die Vorderkammer war beidseits mitteltief, Zellen 1+ (einfach positiv). Die Pupillen waren isokor und rund. Die Pupillomotorik war intakt. Die Iris war reizfrei. Die fundoskopische Untersuchung in Mydriasis zeigte beidseits randscharfe, vitale Papillen mit einer physiologischen Exkavation sowie eine zirkuläre Netzhautanlage ohne Anhalt für ein Berlin-Ödem oder retinale Degenerationen. Auch sonographisch ergab sich im B‑Bild kein Anhalt für einen intraokulären Fremdkörper. Im Endothelzellcount zeigten sich einzelne Lücken in der Endothelzellschicht (Abb. 3). Die Gesamtanzahl an Endothelzellen war rechts 1430 Zellen/mm^2^ und links 2907 Zellen/mm^2^. Die Hornhautdicke war mit 536 µm seitengleich. In der optischen Kohärenztomographie (OCT) des Vorderabschnittes zeigten sich ebenfalls Defekte im Bereich des Endothels (Abb. 4).

## Wie lautet Ihre Diagnose?

## Therapie und Verlauf

Der Patient wurde unter der Diagnose Contusio bulbi mit Payrau-Raynaud-Syndrom stationär aufgenommen. Es erfolgten die beidseitige Versorgung mit Lochklappen sowie eine regelmäßige Tensiokontrolle. Weiterhin erfolgte eine Lokaltherapie mit Prednisolonacetat Augentropfen (AT), Ofloxacin AT und Cyclopentolat AT. Im Rahmen des stationären Aufenthaltes zeigte sich eine stabile Tensiolage, sodass der Patient am Folgetag wieder entlassen werden konnte. Der endotheliale Befund besserte sich im Verlauf.

## Hintergrund

Bei Sandstrahler entsteht durch Druckluft ein starker Luftstrahl, welcher den Sand mit hoher Geschwindigkeit auf das zu behandelnde Objekt bringt. Er wird häufig zum Säubern von Schmutz, Rost und Farbrückständen auf verschiedenen Oberflächen verwendet. Je nach verwendetem Gerät liegt der Betriebsdruck zwischen 5 und 12 bar. Die beschleunigten Sandkörner können je nach verwendetem Gerät eine Ausstromgeschwindigkeit von 80–240 m/s erreichen. Der Patient konnte zu dem vom ihm verwendeten Gerät keine Angaben bezüglich des Betriebsdruckes oder zu anderen Gerätedetails machen.

Das Payrau-Raynaud-Syndrom wurde 1965 erstmals beschrieben und stellt ein kontusionell bedingtes Hornhautendothelödem dar. Es wird auch als Keratopathia annularis posterior bezeichnet [4]. Ursprünglich wurde davon ausgegangen, dass diese endothelialen Veränderungen Folge eines intraokulären Fremdkörpers sein müssen. Es stellte sich jedoch heraus, dass die Befunde nach einer Contusio bulbi durch kleinste Fremdkörper mit hoher kinetischer Energie entstehen, wobei kein intraokulärer Fremdkörper vorliegt. Die Partikel bewirken durch ihre hohe Aufprallgeschwindigkeit bei kleinem Durchmesser ein Fortleiten der kinetischen Energie bis zum Endothel. Hier kommt es infolge dessen zu einer in der Regel ring- oder kreisförmigen Endotheltrübung mit Zellzerstörung und Zellverlust sowie zu einem davor gelegenen oftmals röhrenförmigen Endothelödem [2, 7]. Weiterhin kommt es zu einem endothelialen Zellverlust, jedoch nur bei schwerem Trauma auch zu einer geminderten Endothelzellanzahl. Die geschädigten Zellen werden durch umgebende gesunde Zellen ersetzt [3, 6]. In der Regel ist das Ödem nach wenigen Tagen rückläufig. Es sind jedoch auch andere klinische Bilder beschrieben, bei welchen über das gesamte Kontrollintervall endotheliale Veränderungen zu finden waren. Elektronenmikroskopisch konnte gezeigt werden, dass ein solches Trauma in einem Verlust der Zell-Zell-Kontakte, einer Schwellung der Endothelzellen sowie irregulären Zellwänden resultiert. In schweren Fällen konnte auch ein Verlust einzelner Endothelzellen gezeigt werden [5, 10]. Im Tiermodell wurde außerdem eine stattfindende Entzündungsreaktion mit Akkumulation von Leukozyten und Fibrin nachgewiesen [1]. Das Payrau-Raynaud-Syndrom ist ein sehr selten beschriebenes Krankheitsbild. Bei einem passenden Verletzungsmechanismus sollte deshalb ein genauer Blick auch auf die Endothelzellen geworfen werden. Nichtsdestotrotz bleibt es wichtig, auch andere potenzielle Folgen einer Contusio bulbi im Blick zu behalten (Tab. 1) und ggf. sogar einen intraokulären Fremdkörper auszuschließen. Da es sich bei dem vorliegenden Verletzungsmuster um sehr kleine Partikel handelt, kann auch trotz der durchgeführten Sonographie ein intraokulärer Fremdkörper nicht vollständig ausgeschlossen werden. Es zeigten sich jedoch auch keine indirekten sonographischen Zeichen für eine penetrierende Augapfelverletzung wie beispielsweise eine Ablatio retinae oder eine Aderhautschwellung. Zusätzlich zu der durchgeführten B‑Bild-Sonographie hätte die Durchführung einer Ultraschallbiomikroskopie am vorderen Augenabschnitt evtl. vorliegende intrastromale Hornhautfremdkörper darstellen können. Andere bildgebende Verfahren (wie beispielsweise eine Computertomographie oder eine Magnetresonanztomographie) haben ein deutlich gröberes Raster, sodass kleinste Partikel nicht sichtbar werden.

**Table 1:** 

Lider/Orbita	Ödem, Lidhämatom, Lideinriss, Orbita(boden)fraktur, Weichteilprolaps, Orbitahämatom
Bindehaut	Hyposphagma, Lazeration
Hornhaut	Erosio, Hämatokornea, Epithelödem, Riss der Descemet-Membran, Descemet-Falten, Payrau-Raynaud-Syndrom
Vorderkammer	Hyphäma, Endothelialisierung der Vorderkammer, Vertiefung des Kammerwinkels, sekundäres Offenwinkelglaukom, posttraumatische Kammerwinkelrezession
Iris und Ziliarkörper	Zyklodialyse, Iridodialyse, Aniridie, Irissphinkterkontusion/-nekrose
Linse	Linsenkapselruptur, Linsen(sub)luxation, Kontusionsrosette, Vossius-Ring, Cataracta traumatica
Glaskörper	Glaskörperprolaps, Glaskörperhämorrhagie, Abriss der Glaskörpergrenzmembran
Retina	Berlin-Ödem, Makulaödem, Retinoschisis, Makulaforamen, Ablatio retinae, Netzhautloch, retinale Blutungen, Netzhautnekrose
Choroidea	Aderhautruptur, Aderhautinfarkt, Aderhautabhebung, Chorioretinopathia sclopetaria, sub- und intrachorioidale Blutungen
Sklera	Bulbusberstung
Nervus opticus	Traumatische Optikusneuropathie, Avulsio nervi optici
Verschiedenes	Einsprengung von Fremdkörpermaterial in okuläres und periokuläres Gewebe, sympathische Ophthalmie

**Diagnose:** Payrau-Raynaud-Syndrom

Typische Manifestationen einer Contusio corneae können eine Hämatokornea bei lange bestehendem großem Hyphäma, Rupturen der Descemet-Membran sowie eine Endothelialisierung der Vorderkammer sein [7, 9]. Mögliche Differenzialdiagnosen zu dem beschriebenen Krankheitsbild stellen angeborene Hornhautdystrophien dar, und auch perforierende oder penetrierende Verletzungen können einen ähnlichen Befund zeigen. Deshalb sind eine genaue Anamnese zum Ablauf des Traumas und die Frage nach bereits bekannten ophthalmologischen Erkrankungen unabdingbar. Weiterhin kann eine Verlaufskontrolle zielführend sein, um die Reversibilität beurteilen zu können. Differenzialdiagnostisch sollte bei Descemet-Leisten oder -rissen auch an einen Buphthalmus (im Sinne von Haab-Leisten) als auch an Zustand nach Zangengeburt (vertikale Anordnung der Läsionen) und an einen akuten Keratokonus gedacht werden. Auch hier helfen eine gezielte Anamnese sowie eine gründliche ophthalmologische Untersuchung.

## Fazit für die Praxis

Isolierte endotheliale Verletzungen im Rahmen einer Contusio bulbi sind selten. Jedoch sollte bei entsprechendem Verletzungsmechanismus (kleine Partikel mit hoher Geschwindigkeit) an das Payrau-Raynaud-Syndrom gedacht werden. Nichtsdestotrotz müssen ein intraokulärer Fremdkörper sowie potenzielle andere Differenzialdiagnosen ausgeschlossen werden.
